# Photopolymerization of Chlorpromazine-Loaded Gelatin Methacryloyl Hydrogels: Characterization and Antimicrobial Applications

**DOI:** 10.3390/gels10100632

**Published:** 2024-09-30

**Authors:** Tatiana Tozar, Simona Nistorescu, Gratiela Gradisteanu Pircalabioru, Mihai Boni, Angela Staicu

**Affiliations:** 1 Laser Department, National Institute for Laser, Plasma and Radiation Physics, 409 Atomistilor, Magurele, 077125 Ilfov, Romania; 2 Department of Biochemistry and Molecular Biology, Faculty of Biology, University of Bucharest, 91–95 Splaiul Independentei, 050095 Bucharest, Romania; 3 Research Institute of the University of Bucharest (ICUB), University of Bucharest, 050095 Bucharest, Romania; gratiela.gradisteanu@icub.unibuc.ro; 4 Academy of Romanian Scientists, Ilfov Street 3, 050054 Bucharest, Romania

**Keywords:** Irgacure 2959, gelatin methacryloyl, chlorpromazine, photopolymerization

## Abstract

This study investigates the synthesis, characterization, and antimicrobial properties of hydrogels synthesized through the UV-pulsed laser photopolymerization of a polymer–photoinitiator–chlorpromazine mixture. Chlorpromazine was used for its known enhanced antimicrobial properties when exposed to UV laser radiation. The hydrogel was formed from a mixture containing 0.05% Irgacure 2959, 10% gelatin methacryloyl, and various concentrations of chlorpromazine (1, 2, and 4 mg/mL). Laser-induced fluorescence spectroscopy was employed to monitor the photoinduced changes of chlorpromazine and Irgacure 2959 during hydrogel formation, providing insight into the photodegradation dynamics. FTIR spectroscopy confirmed the incorporation of irradiated chlorpromazine within the hydrogel matrix, while the release profiles of chlorpromazine showed sustained release only in hydrogels containing 1 mg/mL of CPZ. The hydrogel showed significant antimicrobial activity against MRSA bacteria when compared to that of penicillin. These findings highlight the potential of CPZ loaded during the photopolymerization process into hydrogels as effective antimicrobial agents with sustained release properties, making them suitable for combating resistant bacterial strains.

## 1. Introduction

Chlorpromazine (CPZ) is a well-known antipsychotic drug belonging to the phenothiazine class. Beyond its primary use in psychiatry, CPZ has demonstrated significant antimicrobial properties against a variety of pathogens, including bacteria [1,2], fungi [3,4], and mycobacteria [5].

The antimicrobial effect of CPZ irradiated with a 266 nm laser beam has been studied, revealing significant improvements in its antibacterial properties. When CPZ is exposed to a 266 nm laser beam, it undergoes photochemical reactions that produce new species with potent antimicrobial activities [2,6]. These new products exhibit increased efficacy against various bacterial strains, including Gram-positive and Gram-negative bacteria. For instance, prolonged irradiation of CPZ significantly enhances its antibacterial activity against *Staphylococcus aureus* and *Escherichia coli*, although it does not affect their efflux pumps [7]. Additionally, the irradiated CPZ shows antimicrobial and antibiofilm activities against several Gram-positive bacteria, including methicillin-resistant *Staphylococcus aureus* (MRSA) and *Enterococcus* species, by inhibiting efflux pump activity and inducing cellular membrane lesions [2]. Moreover, the products formed during irradiation also demonstrated activity against mycobacteria, including multidrug-resistant tuberculosis strains, with lower cytotoxicity compared to the unirradiated compound [8]. These studies show that CPZ irradiation with pulsed laser radiation could be a promising approach to improve current antimicrobial treatments and combat antibiotic resistance [6,9].

Hydrogels represent an important progression in drug delivery system development [10]. Their customizable properties, biocompatibility, and ability to provide controlled and continuous release of therapeutic agents make them invaluable in biomedical applications [11].

Photopolymerization is a widely used technique for forming hydrogels, particularly in biomedical applications, where gelatin methacryloyl (GelMa) is a popular choice of material due to its biocompatibility and tunable mechanical properties. Photopolymerization uses UV or visible light to dissociate a co-initiator/photoinitiator into radicals that trigger the bonding of monomers or macromers into a crosslinked network, which forms the hydrogel [12]. The type of light source and photoinitiator needs to be selected accordingly because it impacts the efficiency, cytocompatibility, and mechanical properties of the resulting hydrogels [13,14,15]. This technique is widely used in tissue engineering, drug delivery, and the fabrication of microfluidic devices due to its ability to provide precise spatial and temporal control over the polymerization process [16]. This can be achieved by adjusting the light intensity, exposure time, or photoinitiator concentration [13,17]. Photopolymerization, which typically involves the use of a photoinitiator, such as Irgacure 2959, initiates the crosslinking of GelMa upon exposure to UV light [10,18]. Irgacure 2959 is able to reduce the curing time of hydrogels and allows faster photo-crosslinking [10,19]. Additionally, it shows improved mechanical properties, such as compressive modulus and stability, which help to maintain the integrity of the hydrogel [20,21], and it is considered biocompatible [22]. GelMa hydrogels formed through photopolymerization using Irgacure 2959 have been extensively used in various biomedical applications, including tissue engineering [23], wound healing [24], and drug delivery [10,25].

In our previous studies, Tozar et al. [10] presented the synthesis and characterization of hydrogels obtained after the irradiation of various Irgacure 2959 concentrations and GelMa at 10% *w*/*v* up to 30 min with a 266 nm pulsed laser beam at different energies (0.25, 0.45, 0.75, and 1 mJ). Considering the unreacted Irgacure, swelling behavior, drug release profile, and transparency of the hydrogels, the optimal conditions for hydrogel synthesis were a beam energy of 0.75 mJ and exposure time of 1 min. The end goal of the study was to assess the potential of the obtained GelMa hydrogels as drug delivery systems for irradiated CPZ. A volume of 2 mg/mL solution of CPZ (concentration 2 mg/mL) was previously and separately irradiated (energy 6.5 mJ and time period of 30 min), and loaded afterward into already formed GelMa hydrogels. The irradiation conditions for CPZ were chosen in accordance with Tozar et al. [2], where the 30 min irradiated CPZ presented the best irradiation condition in terms of antimicrobial activity. Consequently, the GelMa hydrogel loaded with CPZ (irradiated independently) was shown to be effective against *Staphylococcus aureus* ATCC 25923 and MRSA.

In contrast, the present study aims to uses the same conditions for GelMa hydrogel synthesis (266 nm laser beam, 0.7 mJ energy) while incorporating the irradiation of CPZ directly during the photopolymerization process of the GelMa hydrogels. By irradiating GelMa–Irgacure 2959–CPZ mixtures with a 266 nm laser at 0.7 mJ and for up to 5 min, the active CPZ photoproducts are directly formed during the photopolymerization process within a GelMa hydrogel matrix. The irradiation time was increased up to 5 min to address the simultaneous absorption of 266 nm radiation by both CPZ and Irgacure, as it is possible that longer exposure times might be necessary for proper hydrogel formation. This approach allows direct encapsulation of the drug into the delivery system and can increase the overall efficacy of the drug delivery system by ensuring that the CPZ photoproducts are uniformly distributed within the hydrogel matrix. In this way, hydrogels can protect the CPZ photoproducts from degradation, ensuring their stability and potency over time.

## 2. Results and Discussion

Three concentrations of CPZ (1, 2, and 4 mg/mL), together with Irgacure 2959 (0.05% *v*/*w*) and GelMa (10% *v*/*w*), were chosen to evaluate the effects of various CPZ concentrations on the photopolymerization process and the antimicrobial properties of the resulting hydrogels. This approach allows the determination of the optimal concentration for maximum antimicrobial efficacy while maintaining the structural integrity of the hydrogel. To simplify the terminology used in this paper, Irgacure 2959 at a concentration of 0.05% will be referred to as Irgacure, and GelMa at a concentration of 10% will be referred to as GelMa. The following formulations are named as follows: Irgacure 0.05%–GelMa 10%–CPZ 1 mg/mL hydrogel will be referred to as hydrogel 1, Irgacure 0.05%–GelMa 10%–CPZ 2 mg/mL hydrogel will be referred to as hydrogel 2, and Irgacure 0.05%–GelMa 10%–CPZ 4 mg/mL hydrogel will be referred to as hydrogel 4.

A volume of 35 μL from each solution was placed into a mold and exposed to a 266 nm laser beam (10 Hz repetition rate). The laser beam diameter was adjusted to match the mold’s inner diameter (0.7 cm), and a beam energy of 0.75 mJ (intensity of 19.7 mW/cm^2^) was used. The solutions were irradiated for 1 min and 5 min, delivering a total dose of 1.18 J/cm^2^ and 5.91 J/cm^2^, respectively.

### 2.1. Absorption Spectroscopy Analysis

**Figure 1:**
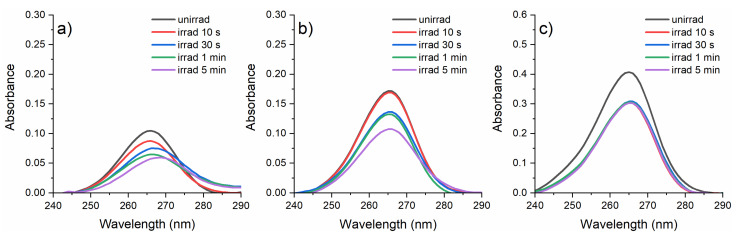
Absorption spectra for CPZ solutions after exposure to 266 nm laser radiation at 0.75 mJ for 5 min at concentrations of (**a**) 1 mg/mL, (**b**) 2 mg/mL, and (**c**) 4 mg/mL.

The absorption spectra of CPZ solutions at the three different concentrations after the irradiation with 266 nm laser beams at 0.75 mJ up to 5 min in the 35 μL molds are shown in Figure 1. The absorption spectrum of unirradiated CPZ was characterized by an absorption band with a maximum at 266 nm. Upon irradiation, the absorption spectrum underwent significant changes only for the 1 mg/mL CPZ solution, where the absorbance began to decrease during irradiation by 30%, and the absorption maximum shifted to longer wavelengths, from 266 nm to 270 nm. This indicates molecular changes in the CPZ molecule due to photochemical reactions induced by the laser irradiation. For the 2 and 4 mg/mL CPZ solutions, there was only a decrease in absorbance (25% for 2 mg/mL, 21.43% for 4 mg/mL), with changes being more pronounced for the 2 mg/mL CPZ solution, suggesting a slower photodegradation of the CPZ molecule at higher concentrations.

Altogether, the absorption spectra showed that the laser-induced photodegradation of CPZ was concentration-dependent, with more substantial photochemical changes occurring at lower concentrations, where the newly formed photoproducts had different absorption characteristics. CPZ is known to undergo several photochemical reactions under UV light [6,26,27], leading to several photoproducts such as CPZ sulfoxide (oxidation of the sulfur atom), desmethyl CPZ (loss of a methyl group from the nitrogen atom), CPZ radical cations (formation of a radical cation through one-electron oxidation, or amino derivatives (hydrolysis of the amide bond leading to various amino derivatives). These photoproducts exhibit different absorption characteristics, typically shifting towards longer wavelengths due to the formation of new conjugated molecules or functional groups [28].

### 2.2. Laser-Induced Fluoresce Analysis

**Figure 2:**
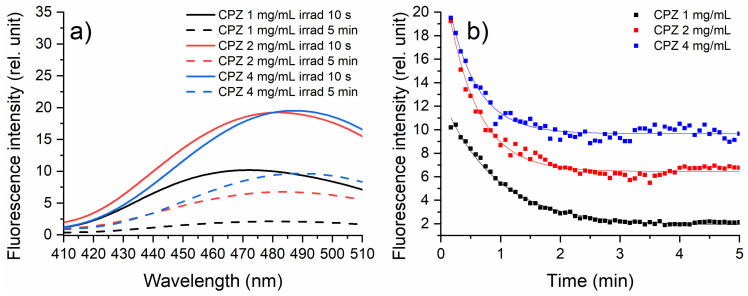
(**a**) LIF spectra of CPZ solutions at concentrations of 1, 2, and 4 mg/mL irradiated with 266 nm for 5 min at an energy of 0.75 mJ; (**b**) fluorescence kinetics profiles for CPZ solutions at concentrations of 1, 2, and 4 mg/mL irradiated with 266 nm for 5 min at an energy of 0.75 mJ.

The laser-induced fluorescence (LIF) spectra and fluorescence kinetics profiles for CPZ solutions at concentrations of 1, 2, and 4 mg/mL irradiated with 266 nm for 5 min at energy of 0.75 mJ are shown in Figure 2. The fluorescence kinetics profile was obtained from the LIF spectrum, recorded every 5 s, and represents the fluorescence intensity of the fluorescence peak. The solution of 1 mg/mL CPZ showed a peak at 465 nm. An increase in fluorescence intensity with increasing CPZ concentration was observed. For CPZ 2 mg/mL and 4 mg/mL, it was noted that in the first 10 s, the fluorescence intensity was similar, indicating that for CPZ 4 mg/mL there was significant absorption of the excitation beam and/or re-absorption of the emission by the sample. At higher concentrations, fluorophore aggregation or re-absorption often occurs, which causes the fluorescence spectrum shape to differ from that of a diluted sample, along with nonlinear concentration behavior [29]. This leads to self-quenching due to proximity interactions, reducing quantum yield and altering fluorescence intensity.

During irradiation, a decrease in fluorescence intensity was observed for all samples (Figure 2b): for 3 min in the case of CPZ 1 mg/mL, 2 min for CPZ 2 mg/mL, and 1.5 min for CPZ 4 mg/mL, after which the intensity remained constant. As CPZ was exposed to radiation, it degraded, leading to a decrease in fluorescence intensity over time. Monitoring this decrease can give insights into the extent and kinetics of photodegradation. Correspondingly, as the concentration of CPZ increased, the wavelength shifted to longer wavelengths, being consistent across both irradiation times (10 s and 5 min). When evaluating the influence of irradiation time, for each concentration, the wavelength was redshifted after 5 min compared to that of 10 s. This indicated that prolonged irradiation further shifts the wavelength, possibly due to increased photodegradation effects or other molecular interactions that occur over time. The intensity of 2 mg/mL CPZ was almost the same as that of 4 mg/mL CPZ when irradiated for 10 s, with a redshift in wavelength for the higher concentration. The redshift of the wavelength with the higher concentration was likely due to the inner filter effect [30], where reabsorption of emitted light causes the observed emission to shift to longer wavelengths.

In Figure 2b, it can be observed that by increasing the concentration of CPZ from 1 to 4 mg/mL, the fluorescence exhibited an increase in intensity for the entire duration of the irradiation process. This was due to the larger number of fluorescent molecules available at higher concentrations, which could absorb more excitation light. The faster decay in fluorescence intensity at lower concentrations suggested that these solutions are more susceptible to photodegradation, possibly due to a higher light dose per number of molecules. Overall, the fluorescence kinetic profiles indicated that the photodegradation rate was inversely related to the concentration of CPZ.

**Figure 3:**
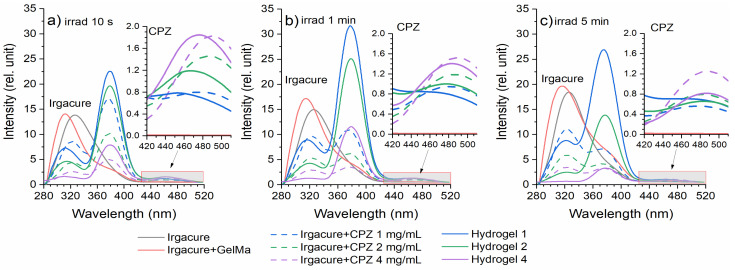
LIF spectra of hydrogels and their constituents recorded following laser irradiation for (**a**) 10 s, (**b**) 1 min, and (**c**) 5 min.

Further, the LIF spectra were registered during hydrogel formation and analyzed to elucidate how laser radiation influences the production of photoproducts and the photopolymerization process. Additionally, for comparison, the LIF spectra for Irgacure, the Irgacure–GelMa mixture, and Irgacure–CPZ (1 mg/mL, 2 mg/mL, and 4 mg/mL) mixtures were measured, and are presented together with hydrogel spectra in Figure 3 for exposures to laser irradiation of 10 s, 1 min, and 5 min. The hydrogel LIF spectra showed the characteristic bands of CPZ and Irgacure, and a new band with intensity maximum at 380 nm.

Irgacure, for an irradiation time of 10 s (Figure 3a), in Irgacure bare solution, exhibited a peak around 330 nm. When Irgacure was mixed with GelMa, the LIF spectrum showed a blue shift of the Irgacure peak position compared to that of Irgacure alone, with slightly higher intensity, probably due to the viscosity of GelMa. As irradiation time increased (1 min—Figure 3b, and 5 min—Figure 3c), Irgacure peak fluorescence intensity increased for both Irgacure and Irgacure–GelMa solutions, with a slight red shift observed for the peak of the Irgacure–GelMa solution. The increased fluorescence under continuous UV irradiation can be attributed to photo-transformation into benzoyl and alkyl radicals [31] or conformational changes, which are species with a higher fluorescence quantum yield.

**Figure 4:**
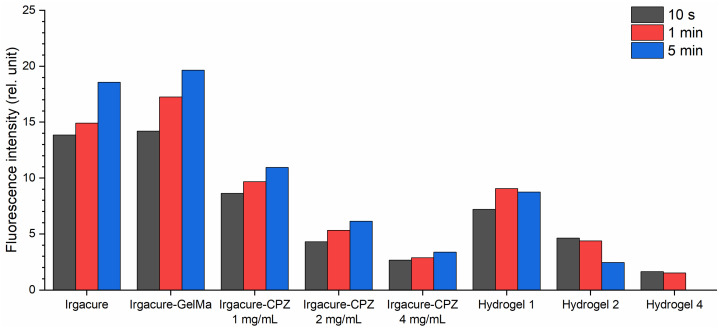
Fluorescence intensity variation of the Irgacure emission peak (330 nm) when Irgacure was exposed to a 266 nm laser beam in different formulations.

When Irgacure was mixed with CPZ, the spectrum combined the emission characteristics of both Irgacure (330 nm) and CPZ (470 nm), along with the formation of a new peak at 380 nm. The new peak at 380 nm was not observed previously by Tozar et al. [10] for Irgacure–GelMa, and probably originated from photochemical-induced reactions between CPZ and Irgacure, and the formation of new chemical species or complexes as a result of laser irradiation. For the Irgacure–CPZ solutions, the Irgacure peak intensity decreased with increasing CPZ concentrations (Figure 3a–c and Figure 4). As the concentration of CPZ increases, there are more CPZ molecules that absorb the excitation light, reducing the available excitation light for Irgacure and thereby lowering its fluorescence intensity. In contrast, at the same CZP concentration, the Irgacure peak intensity increased with longer exposure times, similar to the behavior observed in the Irgacure solution when irradiated alone. As expected, the smallest increase in Irgacure fluorescence intensity as a function of irradiation time was observed when Irgacure was mixed with CPZ 4 mg/mL due to the quenching effect of CPZ molecules on Irgacure fluorescence.

Considering the evolution of the Irgacure peak as a function of CPZ concentration and irradiation time (Figure 4), the Irgacure fluorescence intensity in the hydrogels exhibited a similar pattern with Irgacure–CPZ solutions, but showed a different trend when the exposure time was taken into account. In hydrogel 1, the Irgacure fluorescence intensity increased up to 1 min of irradiation, followed by a slight decrease at 5 min. For hydrogel 2 and hydrogel 4, the intensity of the Irgacure peak decreased continuously throughout the irradiation period, until it disappeared completely for hydrogel 4. The lower Irgacure peak intensity in hydrogels when compared with that of Irgacure–CPZ solution demonstrated consumption of Irgacure in the photo-crosslinking process. The same trend was observed when compared with Irgacure–GelMa. In this respect, the presence of CPZ in the hydrogel introduced competing photochemical and quenching effects that reduced the fluorescence of Irgacure with increasing irradiation time.

**Figure 5:**
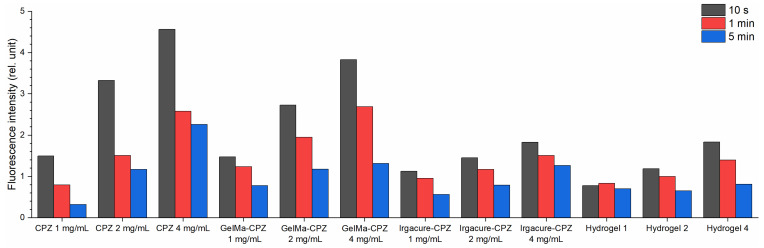
Fluorescence intensity variation of the CPZ emission peak (470 nm) when CPZ was exposed to a 266 nm laser beam in different formulations.

For the CPZ peak at 470 nm, at an irradiation time of 10 s (Figure 3a), the spectra of GelMa–CPZ solutions exhibited an increase in fluorescence intensity with increasing CPZ concentrations. With increasing exposure times (Figure 3b,c), CPZ fluorescence intensity decreased across all the CPZ concentrations used (Figure 5). This showed that CPZ is photodegraded into photoproducts despite the viscous medium created by GelMa.

The fluorescence intensity of the CPZ peak in the GelMa–CPZ solutions exhibited a similar pattern of changes with irradiation time as CPZ alone (Figure 5). However, due to the GelMa matrix, the degradation rate was slowed down, indicating that the overall photodegradation process of CPZ was altered. Further, the fluorescence intensity of the CPZ peak from Irgacure–CPZ formulations (1, 2, and 4 mg/mL) was lower compared to the corresponding concentrations of CPZ alone. This indicates that the presence of Irgacure reduced the fluorescence of CPZ, thus also slowing the photodegradation process, because Irgacure itself absorbed the 266 nm laser beams. The smallest fluorescence intensity of the CPZ peak was also observed in the hydrogel LIF spectra. Since the hydrogel precursor solution consisted of a mixture of GelMa, Irgacure, and CPZ, the fluorescence emission of CPZ was influenced by the interactions with both GelMa and Irgacure. Irgacure competed with CPZ for the absorption of 266 nm photons, while GelMa formed a matrix that encapsulated the photoproducts generated from the irradiated CPZ.

**Figure 6:**
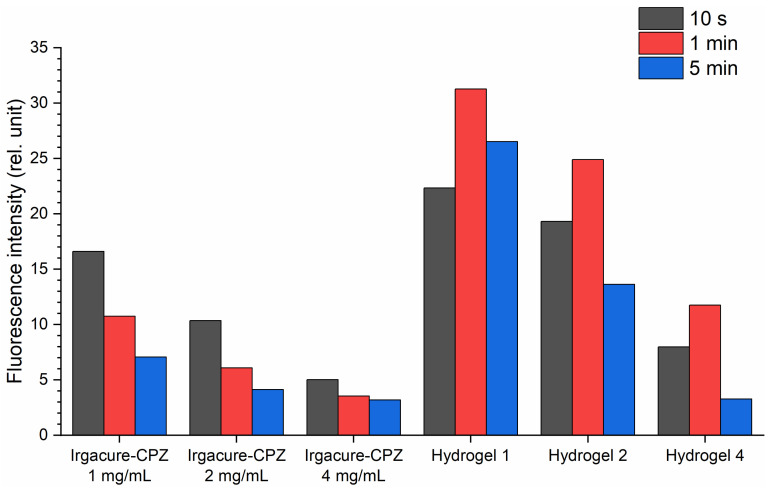
Fluorescence intensity variation of compounds with an emission peak at 380 nm when CPZ and Irgacure were exposed to a 266 nm laser beam in different formulations.

When analyzing the band with a maximum at 380 nm (Figure 6), one can observe that, for the Irgacure-CPZ solution with an increase in laser exposure, the fluorescence intensity followed a similar pattern to that of the CPZ peak (Figure 5) from the same solution, where the fluorescence intensity decreased with longer exposure time. This peak was not present when CPZ and Irgacure were evaluated independently (Figure 3a–c), supporting the hypothesis that the 380 nm peak arises from interactions between CPZ and Irgacure. This behavior suggests that the formation of the compound with a peak at 380 nm was closely linked to CPZ’s photochemical dynamics. In contrast, the fluorescence intensity of the Irgacure peak (Figure 4) increased with irradiation time in the same formulation.

Instead, in the hydrogel formulations, the 380 nm fluorescence peak intensity decreased with increasing CPZ concentrations (Figure 6). This behavior was similar to the evolution of the Irgacure peak in the same formulations, confirming that the compound with a peak at 380 nm was also like the photochemical dynamics of Irgacure, as it was consumed during hydrogel photopolymerization. In addition, the shoulder that appears in the LIF spectra Irgacure–GelMa solution irradiated for 5 min (Figure 3b) demonstrated that a by-product of irradiated Irgacure could be involved in the formation of a 380 nm compound. When comparing the irradiation times, it was observed that the 380 nm peak intensity increased in the first 1 min of exposure, followed by a decrease until the end of the irradiation period. This trend was observed both in the hydrogel (the 380 nm peak investigated) and the Irgacure–CPZ solution (Irgacure peak investigated) only when a 1 mg/mL CPZ concentration was used. These observations highlight how photodegradation, particularly of CPZ, played a critical role in changing the fluorescence dynamics, influencing the relative intensities of the Irgacure and 380 nm peaks over time, and consequently the photopolymerization processes and hydrogel formation.

**Figure 7:**
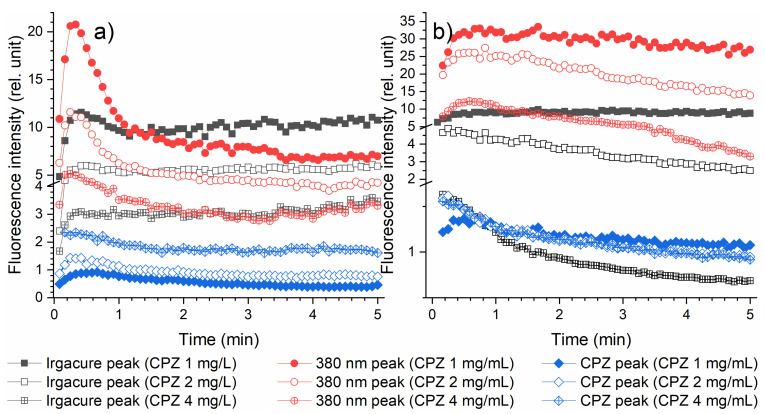
The fluorescence kinetics profile of Irgacure (330 nm peak), CPZ (470 nm), and the compound with a peak at 380 nm in formulations having concentrations of CPZ of 1, 2 and 4 mg/mL in (**a**) Irgacure–CPZ solutions and (**b**) precursor solutions of Irgacure–GelMa–CPZ, which lead to hydrogel photopolymerization; all the samples were irradiated for 5 min with a 266 nm pulsed laser beams at energy of 0.75 mJ.

Figure 7 shows the fluorescence kinetics profiles of Irgacure (330 nm peak), CPZ (470 nm), and the compound with a peak at 380 nm, when CPZ concentration was varied (1, 2, and 4 mg/mL) in formulations such as Irgacure–CPZ solutions and the precursor solution of Irgacure–GelMa–CPZ. When exposed to laser radiation, this precursor solution starts to photopolymerize the GelMa into hydrogel and to transform CPZ into photoproducts in the same time. The fluorescence kinetic profiles represent the fluorescence intensity measured in real time during the irradiation process. In the Irgacure–CPZ solution, the fluorescence intensity of Irgacure and 380 nm peaks increased at the beginning of the irradiation, then decreased, followed by its stabilization. The maximum fluorescence intensity for both Irgacure and the 380 nm peaks occurred at the same times across all initial CPZ concentrations: 25 s for Irgacure and 15 s for the 380 nm peaks. For the CPZ peak, the time required to reach the peak fluorescence intensity was reached after 20 s for CPZ at 1 mg/mL and after 35 s for CPZ at 2 mg/mL. For the initial CPZ concentration of 4 mg/mL in the Irgacure–CPZ solution, from the beginning of irradiation the CPZ peak showed a decrease in fluorescence intensity.

When comparing the CPZ fluorescence peaks, at different initial CPZ concentrations in the Irgacure–CPZ solution (Figure 7a—in blue), it was observed that, for the entire irradiation period, the fluorescence intensity had higher values with increasing CPZ concentration. When analyzing the impact of irradiation time, for initial CPZ concentrations of 1 mg/mL and 2 mg/mL, the CPZ fluorescence initially increased, followed by a decay in intensity. The intensity decay occurred at a higher rate for the 1 mg/mL concentration, indicating a faster photodegradation of CPZ at lower concentration. In contrast, for the Irgacure–CPZ solution with an initial CPZ concentration of 4 mg/mL, there was no initial increase in CPZ fluorescence. Instead, a continuous decrease in fluorescence intensity was observed, but at a slower decay rate compared to the 1 mg/mL and 2 mg/mL concentrations. This suggests a slower photodegradation process due to the higher number of CPZ molecules. Regarding the stabilization times, as the dynamic equilibrium between the processes that influence fluorescence was reached, the time for stabilization became shorter with the increase in the initial CPZ concentration: 275 s for 1 mg/mL, 225 s for 2 mg/mL, and 200 s for 4 mg/mL. These processes could include photodegradation, self-quenching, or complex formation [32,33,34]. The behavior of the stabilization time is due to the high molecular density of CPZ molecules, which enhanced the energy transfer efficiency, leading to faster quenching of excited states and stabilization of fluorescence [35,36].

When comparing the influence of the initial CPZ concentration on Irgacure fluorescence peaks, in the Irgacure–CPZ solution (Figure 7a—in black), it was observed that the overall fluorescence intensity decreased with increasing CPZ concentration. When analyzing the impact of irradiation time, for all initial CPZ concentrations, Irgacure fluorescence initially increased up to 25 s, followed by an intensity decay. The intensity decay occurred at a higher rate for a 1 mg/mL concentration than a 2 mg/mL CPZ concentration, indicating a faster photodegradation of Irgacure. A faster degradation of Irgacure is transposed in a faster photopolymerization of GelMa hydrogels, as the radicals formed during irradiation bind the polymer chains. In contrast, for the Irgacure–CPZ solution with an initial CPZ concentration of 4 mg/mL, a stable Irgacure fluorescence intensity was observed until 200 s, followed by a rapid increase in its intensity. This behavior, combined with the observation that the CPZ peak in the Irgacure–CPZ (4 mg/mL) solution decreased in intensity up to 200 s before stabilizing, suggests that during the first 200 s, the primary process occurring is the photodegradation of CPZ. After 200 s, the increase in fluorescence indicates that the photodegradation of Irgacure becomes the dominant process.

When examining the influence of initial CPZ concentration on the 380 nm fluorescence peaks in the Irgacure–CPZ solution (Figure 7a—in red), it was observed that the overall fluorescence intensity decreased as the CPZ concentration increased. When analyzing the impact of irradiation time, for all initial CPZ concentrations, the 380 nm peak fluorescence initially increased up to 15 s, followed by a decay in intensity. As the initial CPZ concentration increased, the decay rate of the 380 nm peak slowed down due to increased interactions among CPZ molecules. It appears that 200 s was also a significant point for the 380 nm peak in the Irgacure–CPZ (4 mg/mL) solution, as the fluorescence intensity decreased up to 200 s and then began to increase, confirming its existence was linked to both CPZ and Irgacure.

Further, the fluorescence intensity of the same peaks was investigated during the photopolymerization of hydrogel 1, hydrogel 2, and hydrogel 3, and their fluorescence kinetics are shown in Figure 7b.

During the crosslinking of hydrogels 1, 2, and 4, the Irgacure peak (Figure 7b—in black) showed a decrease in overall fluorescence intensity as the initial CPZ concentration increased. In hydrogel 1 (CPZ 1 mg/mL), the fluorescence intensity of Irgacure increased during the first 65 s of irradiation before stabilizing, which is significantly longer compared to the behavior observed in the solutions, i.e., 25 s. In contrast, in hydrogel 2 (CPZ 2 mg/mL) and hydrogel 4 (CPZ 4 mg/mL), the fluorescence intensity of the Irgacure peak began to decrease from the beginning of irradiation, with a fluorescence intensity of the Irgacure peak in hydrogel 2 displaying a faster decay rate than that in hydrogel 4.

For the CPZ peak in hydrogel 1, the fluorescence intensity increased during the first 20 s, then gradually decreased until the end of irradiation. In hydrogels 2 and 4, the CPZ peak fluorescence showed a continuous decline starting from the beginning of irradiation. This behavior is similar to what is observed in the Irgacure–CPZ solution (Figure 7a), but only at a concentration of 4 mg/mL CPZ. The fluorescence decay rate of the CPZ peak is faster in hydrogel 2 compared to hydrogel 4 for up to 150 s of laser exposure, but after this time, the intensities follow a similar trend.

When examining the influence of the initial CPZ concentration on the 380 nm fluorescence peak in the hydrogels (Figure 7b—in red), it was observed that the kinetic fluorescence profile exhibits lower intensities for the entire irradiation time as the CPZ concentration is increased. Independent of the initial CPZ concentration, the 380 nm peak fluorescence increased for the first 40 s of irradiation, followed by a decay. As the CPZ concentration increased from 1 mg/mL to 2 mg/mL, the decay rate of the 380 nm peak was higher. In hydrogel 4 (CPZ 4 mg/mL), the decay rate of the 380 nm fluorescence peak was similar to that observed in hydrogel 2 (CPZ 2 mg/mL) up to 150 s.

The presence of GelMa in the hydrogels introduced additional processes such as crosslinking and photopolymerization, which directly affect the fluorescence kinetics. These processes are absent in Irgacure–CPZ solutions, leading to distinct kinetic profiles between the two environments. In the absence of GelMa, solutions show fluorescence profiles influenced by irradiation and consequent photodegradation. The kinetic profiles represent a combination of direct chemical interactions and structural transformations. The hydrogel matrix limits molecular movement and alter the fluorescence response. The presence of GelMa leads to immediate involvement in polymerization reactions, thus altering the fluorescence profile as crosslinking competes with photodegradation. In solutions, the fluorescence intensity of the Irgacure and 380 nm peaks initially increases, followed by a decline and stabilization, indicating that both peaks are affected by the irradiation and chemical interactions occurring in the solution phase. In hydrogels, the fluorescence intensity of these peaks continuously decreases without significant initial increases. The initial increase in the 380 nm peak observed in solutions indicated the involvement of Irgacure and CPZ, whereas in hydrogels, this increase was smaller because Irgacure was also used in the crosslinking and polymerization reactions that dominated the environment. Moreover, slower decay rates at higher CPZ concentrations were seen in both environments, but hydrogels maintained a generally lower fluorescence due to ongoing photopolymerization.

### 2.3. FTIR Analysis

**Figure 8:**
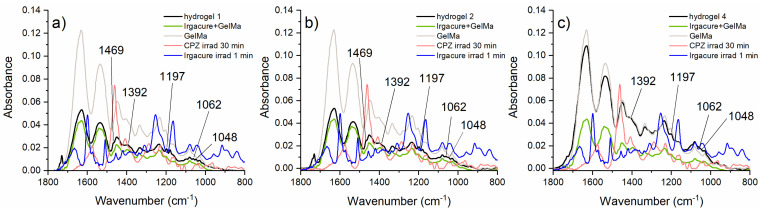
The IR spectra of the Irgacure 0.05%–GelMa 10%–CPZ hydrogels, where the CPZ concentration was (**a**) 1 mg/mL, (**b**) 2 mg/mL, and (**c**) 4 mg/mL.

Further, the overall composition of the samples was examined using FTIR absorption spectroscopy. The IR spectra of the dried hydrogels were recorded in attenuated total reflection mode in the spectral range of 1800–700 cm^−1^ and are presented in Figure 8. These spectra are compared with those of GelMa powder, the 2 mg/mL CPZ dissolved in ultrapure water solution irradiated for 30 min used by Tozar et al. [2], and the Irgacure 0.05% solution irradiated for 1 min. The comparison with the CPZ solution irradiated for 30 min was made to assess whether the photodegradation necessary for achieving the best antimicrobial effect was adequately achieved during the shorter irradiation time used in the hydrogel formation. Additionally, the FTIR spectrum of hydrogels 1, 2, and 4 was compared with that of Irgacure–GelMa (the hydrogel without CPZ), represented by the green line in Figure 8, to determine if the crosslinking density was significantly affected by CPZ.

The IR spectra of the dried hydrogels show a major influence of GelMa, with most of the IR bands of GelMa being present: 1630 cm^−1^—stretching vibration C=O (amide I), 1544 cm^−1^—deformation vibration of C–N–H, 1237 cm^−1^—deformation vibration N-H (amide II), 1450 cm^−1^—deformation vibration C–H, and 1335 cm^−1^—C–N stretching vibration within amide III [10]. The consistent peak positions around 1630 cm^−1^ suggested that the overall crosslinking density of GelMa did not change significantly with CPZ addition. Thus, the FTIR spectrum of hydrogels 1, 2, and 4 showed the presence of the main functional groups of Irgacure–GelMa, indicating the preservation of expected physical characteristics. The IR bands corresponding to the 2 mg/mL CPZ solution irradiated for 30 min can also be observed in the IR spectra of the hydrogels at 1469 cm^−1^ (C–H stretching vibration, CH_2_), 1392 cm^−1^ (C–H deformation vibration, CH_3_), 1197 cm^−1^ (S–O bond stretching vibration, sulfoxide group), 1062 cm^−1^ (in-plane C–H shear deformation vibration), and 1048 cm^−1^ (C–O stretching vibration, phenol group), suggesting the generation of CPZ photoproducts containing phenol and sulfoxide groups. These results mean that the CPZ hydrogels contain the active photoproducts that proved to have antibacterial efficacity in the previous studies [2].

### 2.4. Hydrogel Release Behavior

**Figure 9:**
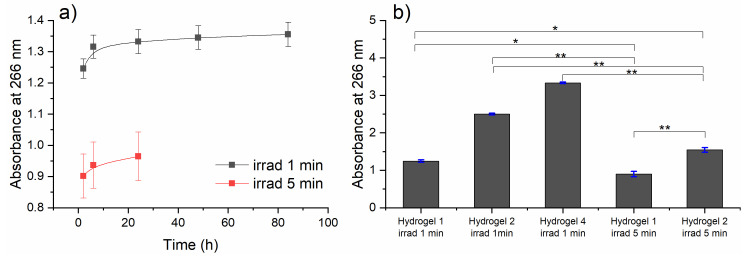
(**a**) Release profiles over time of irradiated CPZ from hydrogel 1 (CPZ 1 mg/mL) photopolymerized for 1 min and 5 min. (**b**) Released irradiated CPZ from hydrogels 1, 2, and 4 within the first 2 h; results are expressed as mean ± standard error from three independent experiments; the data points were analyzed using a *t*-test, showing a statistical significance of *p* < 0.001 between the groups, except for ** *p* < 0.01 and * *p* < 0.05.

The loaded hydrogels were dehydrated before use. Six different hydrogels containing CPZ at 1, 2, and 4 mg/mL were obtained after exposure to a 266 nm laser beam for 1 min and 5 min. They were immersed in 1 mL PBS (phosphate-buffered saline) and incubated at 37 °C for various times (2, 4, 8, 24, 48, and 84 h). To determine the concentration in mg/mL of irradiated CPZ released in the PBS, it is necessary to have a calibration curve with various known concentrations of irradiated CPZ. However, the calibration curve of CPZ irradiated during the hydrogel crosslinking process cannot be obtained, because the specific photoproducts formed during irradiation cannot be directly quantified. As an alternative, the released irradiated CPZ is instead reported based on its absorbance measured at 266 nm (Figure 9). The swelling ratio of these hydrogels could not be determined because, upon immersion in water, the hydrogels immediately began releasing irradiated CPZ, making it impossible to determine their swollen weight.

The release curves of irradiated CPZ demonstrate how the release behavior varies depending on the initial CPZ concentration (1, 2, and 4 mg/mL) and the irradiation duration (1 or 5 min). Among all the hydrogels, only hydrogel 1 (initial CPZ concentration of 1 mg/mL) when exposed to laser radiation for 1 min released irradiated CPZ for periods longer than 24 h (Figure 9a). Hydrogel 1 irradiated for 5 min released irradiated CPZ within only the first 24 h. The rest of the hydrogels, namely, hydrogel 2 (CPZ 2 mg/mL) and hydrogel 4 (CPZ 4 mg/mL), irradiated for both 1 and 5 min, released irradiated CPZ only in the first 2 h. As expected, hydrogel 2 and hydrogel 4 released a higher amount of irradiated CPZ within the first 2 h compared to hydrogel 1 (Figure 9b) due to higher initial concentrations of CPZ. In addition, after 2 h, structural alterations in hydrogels 2 and 4 were observed. This behavior suggested that the structural composition of the hydrogel matrix, influenced by the initial CPZ concentration, played a crucial role in its release behavior.

Moreover, the hydrogels irradiated for longer periods (5 min) showed a lower release compared to those irradiated for shorter durations (1 min). This suggests that the lower release observed in the hydrogels irradiated for 5 min is due to over-crosslinking of the GelMa matrix, resulting in a stiffer structure that more effectively encapsulates the drug, thereby slowing down its release [37,38]. At the same time, it is important to note that longer irradiation significantly affects the photodegradation of CPZ, leading to the destruction of CPZ molecules and their photoproducts, making their detection impossible. These observations highlight the need for careful optimization of irradiation time to balance hydrogel crosslinking and drug release efficiency, ensuring the desired outcome.

### 2.5. Antimicrobial Activity

**Figure 10:**
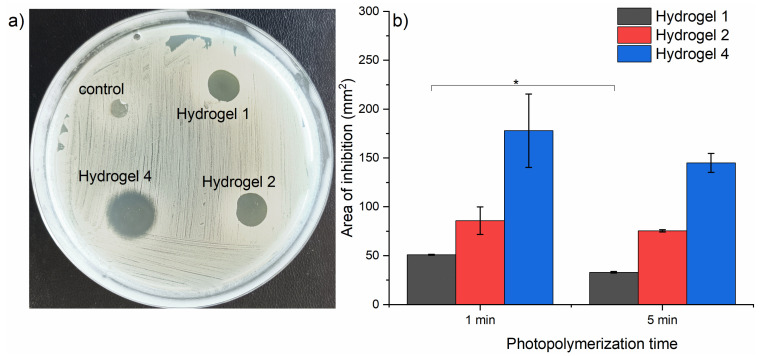
(**a**) Image of inhibition zones for irradiated CPZ released from the control hydrogel, hydrogel 1, hydrogel 2, and hydrogel 4 obtained by 1 min irradiation; the hydrogels were incubated for 18 h at 37 °C together with MRSA. (**b**) Areas of inhibition of irradiated CPZ released from hydrogel 1, hydrogel 2, and hydrogel 4 obtained by 1 min and 5 min irradiation; the areas were extracted from the zone of inhibition images using ImageJ; results are expressed as mean ± standard error from three independent experiments; the data points were analyzed using a *t*-test, showing a statistical significance only between hydrogel 1 resulted from 1 min and 5 min irradiation for * *p* < 0.05.

The antimicrobial activity of the hydrogels was studied on methicillin-resistant *Staphylococcus aureus* (MRSA) using the disc diffusion method and the colony-forming unit (CFU) determination method. The disc diffusion method indicates the susceptibility of the bacterial strain to the loaded hydrogels through the appearance of an inhibition zone (inhibition area) around the hydrogels [39,40]. Figure 10 presents the antimicrobial effects in the form of inhibition zones and inhibition areas for the hydrogels 1, 2, and 4 containing 1, 2, and 4 mg/mL initial CPZ concentrations. Figure 10a) shows the image with the zone of inhibition for irradiated CPZ released from hydrogel 1 (CPZ 1 mg/mL), hydrogel 2 (CPZ 2 mg/mL), and hydrogel 4 (CPZ 2 mg/mL) obtained by 1 min irradiation of Irgacure–GelMa–CPZ (1, 2, and 4 mg/mL) mixtures and compares them with the control hydrogels (Irgacure–GelMa). The inhibition areas from Figure 10b were extracted from Figure 10a using ImageJ software (version 1.54g). The hydrogels were produced through photopolymerization with irradiation times of 1 min and 5 min. For the hydrogel without CPZ, compared to that formed from the irradiation of the Irgacure–GelMa mixture, no inhibition zone was observed (Figure 10a).

The results indicated that the hydrogels containing higher CPZ concentrations (2 mg/mL and 4 mg/mL) showed significantly larger zones of inhibition compared to the 1 mg/mL CPZ hydrogels, reflecting enhanced antimicrobial efficacy against MRSA. Thus, higher drug loading was directly correlated with increased antimicrobial potency. Despite the higher antimicrobial activity, the release behavior of these hydrogels indicated that hydrogels with 2 mg/mL and 4 mg/mL CPZ concentrations primarily released their drug content within the first 2 h. This rapid release pattern occurred due to structural alterations in the hydrogel, which contributed to the large initial inhibition zones but limited the potential for sustained antibacterial effects.

Hydrogels irradiated for 5 min showed smaller inhibition zones compared to those irradiated for 1 min across all CPZ concentrations. This can be attributed to enhanced crosslinking of the hydrogel matrix, which restricts irradiated CPZ release, and the extensive photodegradation of CPZ, reducing its antimicrobial efficacy.

**Figure 11:**
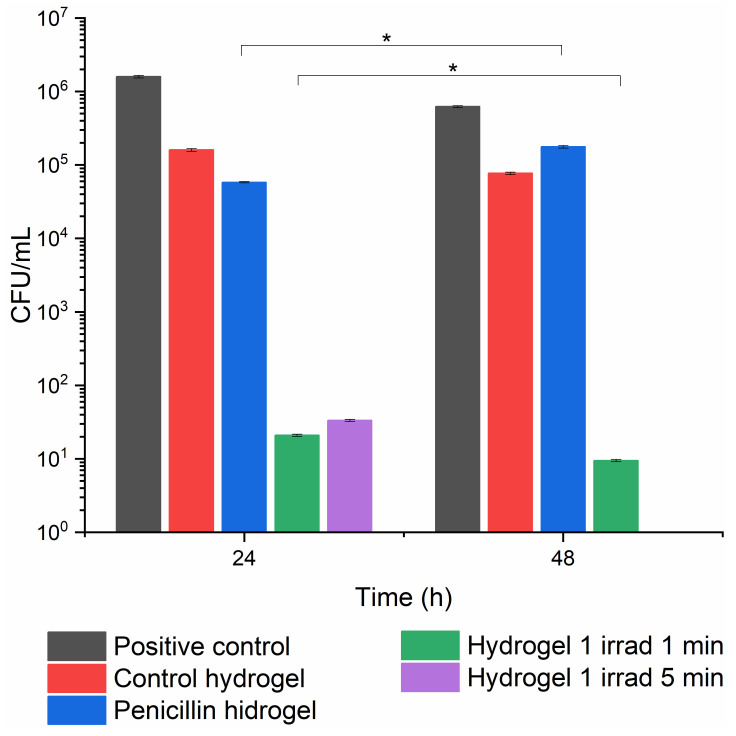
Colony-forming unit (CFU) analysis of MRSA after 24 and 48 h treatment with hydrogels with 1 mg/mL CPZresulted from 1 min and 5 min exposure to laser radiation; the positive control was represented by pure bacterial culture in the absence of hydrogels, the control hydrogels represented hydrogel formed during the photopolymerization of Irgacure–GelMa mixture, and penicillin hydrogel was the control hydrogel loaded with penicillin; results are expressed as mean ± standard error from three independent experiments; the data points were analyzed using a *t*-test, showing a statistical significance between 24 and 48 h for hydrogel 1 and hydrogel loaded with penicillin for * *p* < 0.05.

Further, the CFU method validates the antibacterial effect of the drug-loaded hydrogels in the formation of microbial biofilms [41,42]. The antibacterial effect of hydrogels 1, 2, and 4 was compared with that of a control and penicillin hydrogels. The control hydrogel was the hydrogel formed during the photopolymerization of the Irgacure–GelMa mixture, and the penicillin hydrogel was the control hydrogel loaded with penicillin. In this respect, the adherence of MRSA bacteria to hydrogel 1 resulting from 1 min and 5 min irradiation was assessed using the viable cell count method after 24 and 48 h incubation periods (Figure 11). These hydrogels demonstrated nearly complete inhibition of MRSA colonies after 24 h of treatment. The drastic reduction in the number of bacterial colonies suggested that these hydrogels are highly effective in preventing MRSA adhesion and proliferation during the initial period of exposure. After 48 h, the CPZ hydrogel irradiated for 1 min maintained a very low CFU count, whereas the hydrogel irradiated for 5 min did not release any CPZ after 24 h and was not taken into consideration. Despite this, both hydrogels were far more effective than penicillin and the control hydrogels.

Hydrogels resulting from 1 min laser irradiation showed better performance, maintaining lower CFU counts than those irradiated for 5 min. This aligns with previous observations that shorter irradiation times maintained a better crosslinked structure that optimized the release of CPZ, sustaining its antimicrobial action. The CFU data correlated well with disc diffusion method results, suggesting that 1 mg/mL CPZ hydrogels showed sustained antimicrobial activity over time, even though their inhibition zones were smaller compared to the ones with higher CPZ concentrations. This sustained release helps maintain low bacterial counts over extended periods, making them suitable for longer-term antimicrobial applications.

## 3. Conclusions

This study evaluated both the impact of CPZ at concentrations of 1, 2, and 4 mg/mL on the photopolymerization of hydrogels, and the capability of the hydrogel to have a prolonged and controlled release of the drug and the capability to form antimicrobial photoproducts of CPZ during the same crosslinking process. The assessment focused on absorption spectroscopy, LIF spectra, fluorescence kinetics, FTIR spectra, drug release behavior, and antimicrobial efficiency against MRSA, highlighting that 1 mg/mL concentration is optimal.

The absorption spectra showed that 1 mg/mL CPZ suffered changes in its absorption spectrum upon irradiation with a 266 nm laser beam, indicating photochemical changes. In contrast, 2 mg/mL and 4 mg/mL CPZ exhibited smaller reductions in absorbance and slower photodegradation. The LIF spectra and fluorescence kinetics showed that higher concentrations quench the fluorescence signal due to aggregation and reabsorption effects, also reducing the photodegradation of CPZ. Moreover, the fluorescence decay curves demonstrated that CPZ at the 1 mg/mL concentration displayed the fastest decay, suggesting significant photochemical transformation, which is beneficial for enhancing antimicrobial efficacy. Thus, real-time fluorescence monitoring can provide immediate feedback on the polymerization process, enabling adjustments to UV exposure, light intensity, or component photodegradation. Moreover, the identification of induction periods and stabilization points from direct comparisons of fluorescence profiles helps adjust the photopolymerization conditions for optimal hydrogel formation. Further, the hydrogel showed sustained release only for 1 mg/mL CPZ and a 1 min irradiation time for the photopolymerization process. This prolonged release enhanced the antimicrobial effect against MRSA when compared with that of hydrogel loaded with penicillin. These insights underscore the importance of hydrogel formulation in optimizing the stability, performance, and therapeutic efficacy of irradiated CPZ for antimicrobial applications.

## 4. Materials and Methods

Irgacure 2959 (2-hydroxy-1-[4-(2-hydroxyethoxy) phenyl]-2-methyl-1-propanone), purchased from Sigma-Aldrich (St. Louis, MO, USA), was dissolved in ultrapure water at a concentration of 0.05% *w*/*v*. Gelatin methacryloyl (GelMA), with a gel strength of 300 g bloom and an 80% degree of substitution, was obtained from Sigma-Aldrich and prepared at a concertation of 10% *w*/*v* using ultrapure water as solvent. Chlorpromazine hydrochloride (CPZ, purity of ≥98), was purchased from Sigma-Aldrich and dissolved in ultrapure water. Penicillin G sodium 1,000,000 UI from Antibiotice (Iasi, Romania), used at a concentration of 2 mg/mL, was loaded into the hydrogel resulting from the photopolymerization of the Irgacure–GelMa mixture. This was used as a negative control to compare its antimicrobial activity with that of hydrogels formed during this study. All the powders were used as received without any further purification.

Irgacure 2959 (0.05%)–GelMA (10%) solution was mixed using a magnetic stirrer at 200 rpm for 30 min at a temperature of 70 °C to ensure proper dissolution of each component. Three different concentrations of CPZ (1 mg/mL, 2 mg/mL, and 4 mg/mL) were used together with an Irgacure 2959 (0.05%)–GelMA (10%) mixture to synthesize the hydrogels. All three formulations were tested to determine the optimal concentration of CPZ to obtain the best antimicrobial efficacy while maintaining the structural integrity of the hydrogel. A volume of 35 µL of Irgacure 2959 (0.05%)–GelMA (10%)–CPZ (1 mg/mL, 2 mg/mL, and 4 mg/mL) mixture was placed in a mold and exposed to pulsed laser radiation to obtain the photopolymerization of hydrogels.

The irradiation source was a Continuum Nd laser (10 Hz, 6 ns FWHM) emitting at 266 nm. The solutions were exposed to 266 nm for 1 min and 5 min at a beam energy of 0.75 mJ (I = 19.7 mW/cm²). The mold was 3D-printed from polylactic acid and had an inner diameter of 0.7 cm and a height of 0.1 cm. The diameter of the laser beam was adjusted using dielectric mirrors, lenses, and diaphragms to match the diameter of the mold.

The hydrogels were removed from the mold and immersed in ultrapure water (3 mL) at room temperature to remove precursor residues and to achieve absorption equilibrium (detection of unreacted compounds). They were then dried in a desiccator for 24 h and stored in a desiccator at 4 °C in the dark.

The hydrogels were evaluated using UV-Vis and FTIR absorption spectroscopy, and laser-induced fluorescence spectroscopy. The laser-induced fluorescence (LIF) signal was collected using an optical fiber (M93L02, Thorlabs, core diameter 1500 μm) positioned at 45° to the incident beam and was recorded using a SpectraPro SP-2750 spectrograph (Acton Research, Czerny-Turner configuration, Trenton, NJ, USA) coupled with a PIMAX 1024RB ICCD camera (Princeton Instruments, Trenton, NJ, USA). A Perkin Elmer spectrophotometer (Waltham, MA, USA), model Lambda 950, was used to record absorption spectra with a resolution of 1 nm. The optical path of the spectrophotometric cell used was 0.01 mm. The Nicolet iS50 FTIR spectrometer (Thermo Fisher Scientific, Waltham, MA, USA) was used to record the IR spectra of the aqueous solutions, dried hydrogels, and powder forms. The IR spectra of the solution samples were recorded in transmittance mode between 1800 and 700 cm^−1^, at a resolution of 4 cm^−1^ and an average of 32 spectra. The dried hydrogels and powder form samples were measured in attenuated total refraction mode, equipped with a ZnSe crystal, in the spectral range of 1800–700 cm^−1^, with a resolution of 4 cm^−1^ and an average of 16 spectra.

The antimicrobial activity of the hydrogels loaded with CPZ irradiated during the photopolymerization process was investigated on methicillin-resistant *Staphylococcus aureus* (MRSA) by the disk diffusion method [39,40] and viable cell count method [41,42]. The disk diffusion method was conducted by inoculating with a bacteria suspension on the surface of the agar plate. Afterward, instead of a disc, the hydrogels were directly applied to the agar plate and incubated together at 37 °C for 18 h. The hydrogels were in direct contact with the bacterial culture. The susceptibility of the bacterial isolates to each hydrogel was subsequently quantified by measuring the inhibition zone areas using ImageJ software. Further, the viable cell count method was used to determine the bacterial adherence to the hydrogels. A bacterial suspension of 10^6^ CFU/mL was prepared in phosphate buffer solution (PBS). In this assay, both positive and negative controls were included to ensure the validity of the results. Two positive controls were used, one represented by pure bacterial culture in the absence of hydrogels, and the other represented by hydrogel formed during the photopolymerization of Irgacure–GelMa mixture and named the control hydrogel. The negative control was a penicillin hydrogel, represented by a control hydrogel loaded with penicillin. The control hydrogel, hydrogel loaded with penicillin, and the hydrogels with 1, 2, and 4 gm/mL CPZ were incubated at 37 °C for 24 and 48 h together with 1 mL of bacterial suspension. Following that, the hydrogels with attached bacteria were extracted, immersed in 1 mL PBS, and vortexed. Serial dilutions of the samples were plated onto Plate Count Agar and incubated overnight at 37 °C. The bacterial quantity in the initial sample was determined by counting the colonies on the plates and factoring in the dilution ratio.

## Data Availability

All data and materials are available on request from the corresponding author.
